# Ictal semiology in supplementary motor area and pre‐supplementary motor area epilepsy: A systematic review and meta‐analysis

**DOI:** 10.1002/epd2.70137

**Published:** 2025-11-24

**Authors:** Simona Buonocore, Marianna Pommella, Alessandra Bettiol, Salvatore De Masi, Carmen Barba

**Affiliations:** ^1^ Department of Neurosciences, Psychology, Drug Research and Child Health (NEUROFARBA) University of Florence Florence Italy; ^2^ Neuroscience and Human Genetics Department Meyer Children's Hospital IRCCS Florence Italy; ^3^ Department of Biomedical, Experimental and Clinical Sciences ‘Mario Serio’ University of Florence Florence Italy; ^4^ Scientific Directorate Meyer Children's Hospital IRCCS Florence Italy

**Keywords:** anatomo‐clinical correlation, epilepsy surgery, ictal semiology, stereo‐EEG, systematic review

## Abstract

We conducted a systematic review and meta‐analysis of the ictal semiology associated with supplementary motor area (SMA) and pre‐supplementary motor area (pre‐SMA) epilepsy, to summarize current knowledge of related anatomo‐clinical correlations in the context of presurgical evaluation. We conducted the review and reported its results according to the Preferred Reporting Items for Systematic Review and Meta‐Analysis statement (PRISMA). We searched PubMed and Embase using relevant keywords related to the SMA and pre‐SMA localization, seizure semiology, and scalp electroencephalography (EEG) or stereo‐EEG. The risk of bias was evaluated using the QUADAS2 score. Twenty articles were included, with extractable data from 37 patients. We analyzed the included studies and extracted data on the presence of 12 different symptoms. We then performed a meta‐analysis of the proportion of patients with each symptom. The most frequently reported ictal feature in SMA epilepsy was asymmetric tonic posturing, observed in 47% of cases. Automatisms (25%) and versive seizures (23%) were also common, while loss of consciousness occurred in 19% of patients. Sensory phenomena (11%) and speech arrest/inhibition (10%) were less frequent. Other features, including symmetric tonic posturing, elementary motor signs, hyperkinetic patterns, affective phenomena, grimacing, and negative motor phenomena, were rarely observed. Little evidence is available on the distinct involvement of the pre‐SMA. Although asymmetric tonic posturing appears to be the most common feature of SMA epilepsy, it occurs in fewer than 50% of patients and the level of evidence of this association remains low. The same semiological feature may result from the rapid propagation to adjacent or connected regions; hence, semiology should always be interpreted in the context of a multimodal evaluation. Stereo‐EEG investigation remains crucial when EEG and imaging are inconclusive or conflicting.

**Key points**

- Diagnosing SMA epilepsy is challenging due to overlap with other frontal seizures and non‐localizing EEG findings.
- Asymmetric tonic posturing is the most frequent sign of SMA epilepsy, though it is observed in fewer than half of the patients.
- Automatisms, version, and speech arrest are common yet less specific to SMA epilepsy.
- Ictal semeiology may reflect early propagation to adjacent cortical regions and should always be interpreted in the context of a multimodal evaluation.
- Stereo‐EEG is crucial for localizing the Epileptogenic Zone in SMA and pre‐SMA, when MRI and scalp EEG are inconclusive.

## 1 INTRODUCTION

The supplementary motor area (SMA), located on the medial surface of the superior frontal gyrus, plays a central role in motor planning and initiation, particularly for complex, sequential, and bilateral movements. It is anatomically subdivided into the rostral pre‐SMA and the caudal SMA‐proper, two subregions which exhibit distinct patterns of connectivity and functional specialization. The identification of seizures originating from the premotor cortex dates to the early 20th century. A landmark in this field is the work of Penfield and Jasper in 1954, who provided the first detailed clinical description of seizures involving the SMA.^1^ Their seminal observations described tonic limb posturing, adversive movements, and vocalizations as key semiological features. Notably, none of the patients in their series achieved seizure freedom following SMA resection, leading the authors to hypothesize that SMA was involved in symptom generation but not necessarily represented the primary epileptogenic zone (EZ). The concept of “SMA seizures” as emerging from these early observations, was further delineated in subsequent decades especially through stereoelectroencephalography.^2^, ^3^, ^4^ Although not included in the initial ILAE classification,^5^ SMA seizures were later described in 1989^6^ as a subtype of frontal lobe seizures characterized by “postural, focal tonic with vocalization, speech arrest, and fencing postures.^6^”

Over time, the ictal semiology of SMA seizures has been mainly reported as featuring asymmetric tonic posturing, involving both upper and lower limbs^4^ often associated with loss of consciousness.^7^ Seizures are typically brief, with an abrupt onset and termination. They mainly occur during sleep, which can lead to the misdiagnosis of “sleep disorders.”^8^ However, although less frequently, negative phenomena, including speech inhibition and motor arrest, can also characterize seizures arising from the supplementary motor area.^9^ Seizures may either remain confined to the SMA or demonstrate rapid secondary bilateralization, thus contributing to the heterogeneity of their clinical presentation. In this context, Ikeda et al. proposed a distinction between “SMA seizures,” defined as seizures that may either originate within the SMA or rapidly propagate to it from adjacent regions, and “SMA epilepsy,” which refers to a well‐demarcated epileptogenic zone confined to the SMA.^10^

However, distinguishing between “SMA seizures” and “SMA epilepsy” remains challenging, despite advances in neuroimaging and neurophysiological techniques. We thus performed a systematic review of the ictal semiology of SMA and pre‐SMA seizures with the view to summarize the state‐of‐the‐art of anatomo‐clinical correlations in the field and help guide interpretation of ictal semiology within the framework of pre‐surgical evaluation.

## 2 MATERIALS AND METHODS

### 2.1 Search method and eligibility criteria

We conducted a systematic review of the published evidence and reported its results according to the Preferred Reporting Items for Systematic Review and Meta‐Analysis (PRISMA) statement.^11^ PubMed and Embase databases were searched for papers published in English, covering publication up to December 2023. The search strategy included terms related to the anatomical origin (“supplementary motor area” OR “pre‐supplementary motor area”) combined with seizure‐related terms (“epilep*” OR “seizure”) and additional terms related to diagnosis and management (“surgery” OR “EEG” OR “video” OR “semiology”). Boolean operators were used to optimize sensitivity and specificity.

We selected studies published as papers in peer‐reviewed journals, with an abstract available. We considered as eligible only primary studies on adult and pediatric (<18 years) patients undergoing presurgical evaluation and/or epilepsy surgery whose epileptogenic zone was localized to the SMA or pre‐SMA (“SMA or pre‐SMA epilepsy”). We included studies of the following criteria:

- With at least 12 months of follow‐up after surgery;
- Conducted on at least one patient (informative case reports were deemed eligible as they could provide valuable insights into clinical presentation);
- In which anatomo‐electroclinical correlations were explicitly investigated, with patients who underwent invasive EEG monitoring and/or presented with a clearly identifiable MRI lesion;
- Indicating seizure outcome if surgery was performed (Engel classification).^12^

In addition, we included articles meeting the inclusion criteria from the bibliography of the papers primarily selected through PubMed and Embase.

Two independent reviewers (SB and MP) screened titles, abstracts, and full‐text articles for eligibility criteria. Two additional reviewers (CB and SdM) resolved disagreements at the full‐text screening phase and the data abstraction phase.

### 2.2 Data extraction

For each selected publication, we extracted the number of reported patients, the proportion of patients with the epileptogenic zone hypothesized in the SMA and pre‐SMA, and all data informing on anatomo‐clinical correlations. We evaluated the risk of bias of each publication using a QUADAS2‐adapted^13^ assessment at the level of each selected publication. We further assessed our level of confidence in the reported epileptogenic zone according to a recently developed method.^14^ The latter is based on the availability and findings from MRI, intracerebral EEG and post‐operative outcome, and distinguishes four levels of evidence: very high, high, moderate, and low. When multiple sources of information were available and suggested different confidence levels, priority was given to post‐operative outcome over intracerebral EEG findings, and to intracerebral EEG findings over MRI data. For each selected paper, we indicated the proportion of patients falling into each of the above‐mentioned confidence categories. The summary of evidence was then assessed using the GRADE system. Detailed information regarding the QUADAS‐2 assessment, the summary of evidence and the level of confidence assigned to the localization of the epileptogenic zone can be found in Supplementary Material (Data S1).

### 2.3 Statistical analysis

Descriptive statistics were used to summarize patient demographics and surgical outcomes. Seizure semiology features were classified based on the clinical descriptions reported in the selected studies. In line with the most recent classifications provided by the International League Against Epilepsy,^15^, ^16^ twelve ictal symptoms were retrospectively identified from the available data: asymmetric tonic posturing, symmetric tonic posturing, elementary motor signs in combination (unilateral tonic posture followed by unilateral clonic activity), automatisms (oroalimentary automatisms, gestural automatisms, vocal automatisms), version, speech inhibition, other negative phenomena (atonia, palilalia), hyperkinetic pattern, facial grimacing, sensory phenomena (somatosensory‐paresthesia), affective phenomena (fear, anxiety), and loss of consciousness.

We analyzed the included studies and extracted data to assess the proportion of patients presenting with at least one of the 12 symptoms. We then conducted a meta‐analysis of the prevalence of each symptom reported across studies, based on the total number of patients with a confirmed SMA epilepsy. When the number of events was zero, the software used made a continuity correction, adding .5 to event counts and sample sizes.^17^ Heterogeneity between studies was assessed using the chi‐squared test and quantified using the I‐squared parameter. All analyses were performed with Stata version 16.

## 3 RESULTS

### 3.1 Study selection and characteristics

**FIGURE 1:**
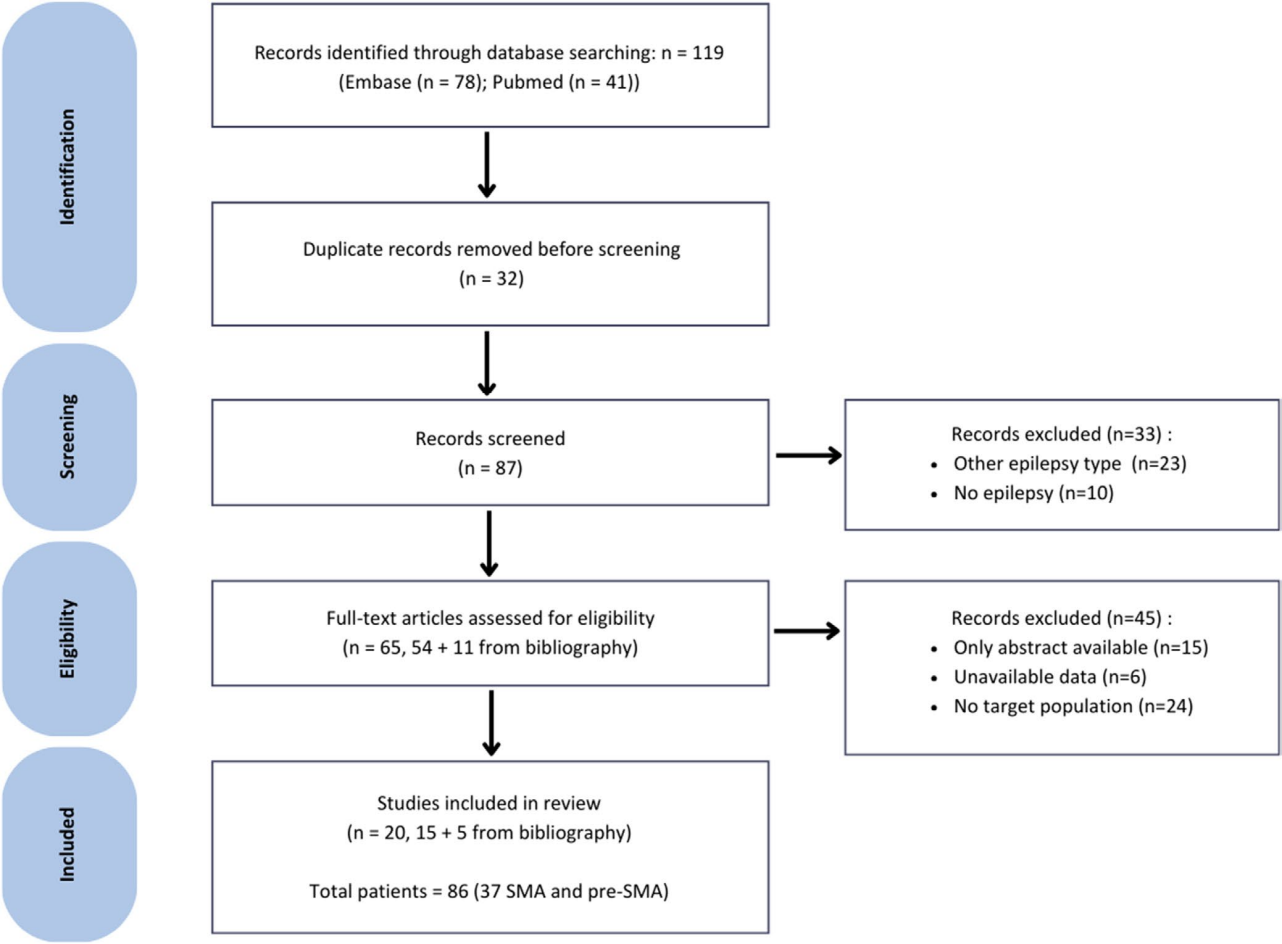
PRISMA flow diagram of the study selection. *n*, number of records at each step; pre‐SMA, pre‐supplementary motor area; SMA, supplementary motor area.

A total of 41 records were identified through PubMed and 78 through Embase. Following the screening and eligibility assessment, 20 studies met the predefined inclusion criteria and were included in the final analysis. The detailed selection process is outlined in the PRISMA flow diagram (Figure 1). Three articles included more than 10 patients, and nine articles were case reports leading to a total number of 86 patients, 37 of whom had SMA or pre‐SMA involvement. The mean number of patients per study was two (1–26), the mean number of patients with SMA/pre‐SMA involvement was one (1–7), the median percentage of patients explored using Stereo‐EEG and operated on was 100% (see Tables 1 and 2 for all the details). There were no case–control studies; all studies reported random cases referring to a specific clinical sign in relation to the EZ localization to SMA or pre‐SMA. Confidence in the localization of the epileptogenic zone was rated as “high” or “very high” in almost all cases (91.9%). However, according to the GRADE approach, the overall certainty of the association between clinical signs and the SMA origin is low to very low, due to the predominance of case reports, small sample sizes, methodological heterogeneity, and selection bias.

**TABLE 1:** Detailed information on individual study results.

Study	No. of patients	% MRI positive	Adults (A), Children (P)	% iEEG	% operated	% class Ia ≥1 year	Confidence EZ (%very high/high/moderate/low)
Shuichiro Neshige et al. 2019	1	100	1A‐0P	100	100	100	100/0/0/0
Van Tri Truong et al. 2013	1	0	1A‐0P	100	100	100	100/0/0/0
Akio Ikeda et al. 1999	1	100	1A‐0P	100	100	0	0/100/0/0
Takeshi Inoue et al. 2021	1	0	0A‐1P	100	100	100	100/0/0/0
S. Meletti et al. 2002	1	100	1A‐0P	0	100	100	100/0/0/0
Mitsumasa Fukuda et al. 2023	1	100	1A‐0P	100	100	100	100/0/0/0
B. C. Jobst et al. 2000	7	14–43	4A‐3P	100	100	71.4	71.4/27.6/0/0
K. M. Smith et al. 2022	1	0	1A‐0P	100	100	0	0/100/0/0
Kovac et al. 2012	1	0	1A‐0P	100	100	0	0/100/0/0
Yang‐Je et al. 2009	1	100	1A‐0P	0	0	0	0/100/0/0
Cukiert et al. 2001	1	0	1A‐0P	100	100	0	0/100/0/0
Ciurea et al. 2015	1	0	1A‐0P	100	100	100	100/0/0/0
Ikeda et al. 2002	1	100	1A‐0P	100	100	50	0/100/0/0
Morris et al. 1988	3	100	3A‐0P	100	33	100	33/66/0/0
Barba et al. 2005	2	100	2A‐0P	100	100	100	100/0/0/0
Baumgartner et al. 1996	5	60	4A‐1P	100	100	40	60/0/40/0
Job et al. 2014	3	33	1A‐2P	100	100	33	66/33/0/0
Nobili et al. 2003	1	0	1A‐0P	100	100	100	100/0/0/0
Scholly et al. 2017	1	100	0A‐1P	100	100	100	100/0/0/0
Takayasu et al. 1993	3	100	3A‐0P	100	100	33	66/0/33/0

*Note*: Patient demographics, imaging findings, surgical outcomes, and confidence levels in identifying the epileptogenic zone (EZ) across the 20 studies included in the review. It includes data on the number of included patients (adults or pediatric), percentage of positive MRI, percentage of patients undergoing iEEG, percentage of patients operated on, percentage of patients on Engel Class Ia at 1 year, and the confidence in EZ identification (rated as very high, high, moderate, or low).

Abbreviations: EZ, epileptogenic zone; iEEG, invasive EEG.

**TABLE 2:** Aggregated data across the selected studies.

	No. of patients	% MRI positive	% adults	% iEEG	% operated	% class Ia ≥1 year	Confidence in the EZ
Min	1	0	0	0	0	0	Moderate
Max	7	100	100	100	100	100	Very high
Median	1	80	100	100	100	85.7	NA
Total	37	21 (56.8%)	29 (78.4%)	35 (94.6%)	34 (91.9%)	22 (59.4%)	Moderate 8.1%, High 29.7% Very High 62.2%

*Note*: Aggregated data across the 20 selected studies: median values for the number of included patients (adults or pediatric), percentage of positive MRI, percentage of patients undergoing iEEG, percentage of patients operated on, percentage of patients on Engel Class Ia at 1 year, and the confidence in EZ identification (rated as very high, high, moderate, or low).

Abbreviations: EZ, epileptogenic zone; iEEG, invasive EEG.

Among the 37 patients included in the study, 8 (21.6%) were children (<18 years of age). MRI was unrevealing in 16 patients (43.2%), and invasive EEG monitoring with stereo‐EEG or subdural grids was performed in 35 patients (94.6%). Epilepsy surgery was carried out in 34 patients (91.9%), resulting in seizure freedom (Engel class Ia) in 22 cases (59.4%) after a follow‐up of at least 12 months. Two of the three remaining patients were included because Stereo‐EEG findings indicated that their seizures originated in the SMA. The remaining patient presented an encephalomalacic lesion in the left superior frontal gyrus; an analysis using subtraction ictal‐interictal SPECT co‐registered to MRI revealed a significant increase in perfusion involving the SMA.

### 3.2 Seizure semiology

The main semiological features and their characterization are indicated in Table 3. A meta‐analytic approach was employed to estimate the overall prevalence of each reported ictal manifestation.

**TABLE 3:** Type of ictal symptoms reported in SMA epilepsy and their pooled prevalence from the meta‐analysis.

Ictal symptom	No. of patients	Pooled prevalence (%)	More at onset or during propagation
Asymmetric tonic posturing	17	47	Onset
Automatisms	11	25	Onset
Version	11	23	Onset
Loss of consciousness	9	19	Propagation
Sensory phenomena	8	11	Onset
Speech inhibition	7	10	Onset
Symmetric tonic posturing	5	5	Onset
Grimacing	5	5	Onset
Elementary motor signs	5	4	Onset
Other negative phenomena	4	1	Propagation
Hyperkinetic pattern	3	1	Onset
Affective phenomena	3	1	Propagation

Asymmetric unilateral tonic posturing emerged as a prominent and characteristic feature of SMA epilepsy, with a pooled prevalence of 47%, nearly half of the patients reported, and a moderate heterogeneity which suggests some variability across studies (*I* ^2^ = 33.49%, *p* = .07). Head and/or eye version and automatisms were observed in approximately one‐fourth of the reported patients, respectively 23% and 25% of patients. The studies were homogeneous regarding versive symptoms (*I* ^2^ = .00%, *p* = .47), with a low heterogeneity when reporting automatisms (*I* ^2^ = 14.19%, *p* = .28). Although loss of consciousness is not traditionally considered a hallmark of seizures originating from SMA, it was reported in 19% of cases; the heterogeneity was moderate, reflecting some variability across studies but no clear inconsistency in the overall pattern (*I* ^2^ = 30.37%, *p* = .10).

Speech inhibition was reported in 10% (*I* ^2^ = .51%, *p* = .45) and sensory phenomena in 11% of the population (*I* ^2^ = .00%, *p* = .59). Conversely, features such as hyperkinetic pattern, symmetric tonic posturing, other elementary motor signs, grimacing, affective phenomena and other negative phenomena (e.g., atonia) were rare (<5%). For a comprehensive overview, see Figures S1–S12.

## 4 DISCUSSION

We conducted a systematic review of the semiological spectrum of SMA and pre‐SMA epilepsy to provide insights into the localization of EZ for presurgical evaluation. Identifying seizures truly originating from these brain regions poses significant clinical challenges, primarily due to the semiological overlap with other frontal lobe seizure types. Accurate delineation of seizure onset can be particularly challenging due to the frequent lack of localizing interictal or ictal abnormalities on scalp EEG; the deep, mesial position of the SMA often results in nonspecific or falsely localized EEG patterns.^7^, ^18^ Nearly all patients from the studies included in our review (92%) underwent invasive EEG evaluation, most commonly stereo‐EEG, to confirm that the EZ was located in SMA and guide surgical resection. Intracranial recordings have emerged as an essential modality for enabling differentiation between seizures truly originating in the SMA and those propagating from adjacent frontal regions.^19^ As shown in this review, stereo‐EEG has been used in patients with or without MRI‐visible structural abnormalities, when scalp EEG and/or semiological features were inconclusive or conflicting.^20^

In this context, ictal semiology represents the primary clinical clue suggesting a possible SMA EZ, to be subsequently integrated with EEG and imaging data. Our meta‐analysis highlights that asymmetric tonic posturing is the most prevalent manifestation, reported in nearly half of the patients, and typically appearing at seizure onset. This finding aligns with extensive literature describing SMA as the origin of this specific ictal pattern. From early seminal accounts by Penfield and Jasper^1^ to more recent studies, this feature has been repeatedly recognized as a key marker distinguishing between SMA seizures and SMA epilepsy.^10^ For instance, Sitthinamsuwan et al. suggested that patients with seizures originating in the SMA, confirmed via stereo‐EEG, exhibited unilateral or bilateral asymmetric tonic posturing significantly more often than those with extra‐SMA onset, who more commonly presented with symmetric tonic posturing.^21^ Complex gestural automatisms have been frequently reported in seizures originating from frontal lobe areas.^6^, ^22^ Among these, the SMA, and especially the pre‐SMA,^23^, ^24^, ^25^, ^26^ may be involved in the generation of stereotyped repetitive movements as confirmed in our meta‐analysis; however, due to the limited number of observations, we could not attribute a specific type of automatisms to either the SMA or pre‐SMA. In general, the differentiation between SMA and pre‐SMA epilepsy was possible only in a few studies.

The SMA has been considered the epileptogenic zone in a minority of patients with contralateral versive manifestations, which have been more frequently associated with other frontal areas such as the dorsolateral premotor cortex.^27^, ^28^ However, the relatively high frequency of this ictal manifestation in our meta‐analysis suggests that the SMA may play a direct role in its generation, rather than merely being involved via spread from other cortical regions.^29^, ^30^, ^31^ Actually, all three aforementioned manifestations, that is, unilateral/bilateral asymmetric tonic posturing, automatisms and version, were mostly reported at seizure onset, thus highlighting their possible role as early localizing signs in SMA epilepsy.

In our meta‐analysis, loss of consciousness was observed in a small proportion of patients. However, there is potential bias in the available data. Patients were not uniformly assessed for consciousness /responsiveness, and their presence or absence was not consistently reported across the included studies.

Ictal speech inhibition, though less frequent, is a well‐documented manifestation in SMA epilepsy, recognized since the earliest ILAE classifications.^6^ It may reflect an ictal disruption of motor execution,^9^ consistent with findings from electrical cortical stimulation studies. These studies have demonstrated the critical role of SMA and pre‐SMA in both the initiation and inhibition of speech, with stimulation often resulting in speech arrest without affecting consciousness.^32^, ^33^, ^34^, ^35^, ^36^, ^37^ Other negative phenomena, such as atonia, were rarely reported in our systematic review but may share a common functional substrate with speech inhibition. They could reflect a transient disruption of motor output pathways, particularly during early stages of seizure activity before recruitment of adjacent motor areas.^38^, ^39^

In our cohort, 11% of patients experienced sensory phenomena prior to the onset of motor symptoms. Sensory auras have already been described in association with SMA epilepsy and may present as nonspecific cephalic sensations, paresthesia, or a sense of impending movement, typically diffuse and poorly localized.^40^, ^41^ Sensory phenomena, such as numbness, dizziness, tingling, have been reported during cortical stimulation studies of the SMA, leading Lim et al.^36^ to conclude that the term “supplementary sensorimotor area (SSMA)” represents a more appropriate expression to identify this cortical region. Other features, such as hyperkinetic movements, elementary motor signs, grimacing, affective features appear uncommon in our systematic review, likely because they are not distinctive for SMA involvement. In particular, hyperkinetic seizures have been widely described in association with frontal lobe epilepsy,^42^, ^43^ especially the ventromedial prefrontal cortex^26^ with a rapid propagation to motor and premotor areas.

Another ictal motor manifestation observed in a small subset of patients in our systematic review was represented by clonic jerks following focal tonic manifestations. Seizures originating from the SMA are usually associated with sustained tonic muscle contractions, whereas clonic jerks are more characteristic of seizures arising from the primary motor cortex. The emergence of clonic activity after tonic manifestations in these patients likely reflects the propagation of ictal discharges from the SMA to the primary motor area.^44^

Although fear and other affective features are more commonly associated with seizures originating from the limbic and paralimbic regions, they have also been reported in a few (<5%) of the patients included in our meta‐analysis. This manifestation is likely related to the rapid propagation of ictal activity to the anterior cingulate cortex and orbitofrontal regions.^45^ Within this framework, facial grimacing, especially ictal pouting or chapeau de gendarme, has been described as a facial expression conveying a range of negative emotions. Although reported in a few patients in association with SMA seizures, this ictal feature has been interpreted as a behavioral manifestation rather than a purely motor phenomenon. It may reflect the activation of affective networks within the medial frontal regions, including the anterior cingulate and the medial prefrontal and premotor cortex.^45^, ^46^

### 4.1 Limitations of the review

The main limitations of our systematic review included the low number of observations, all retrospective, and the high number of case reports. In addition, due to the inherent characteristics of systematic reviews assessing semiological features in retrospective cases, a uniform evaluation of seizure onset and ictal semiology cannot be assumed across all reports. Finally, due to the specific focus of this review, we did not examine the role of frontal networks as in some previous studies, which were not included in our study because they did not allow separate analysis of patients with SMA epilepsy. For instance, Bonini et al.^26^ expanding upon previous investigations from the same group into the clinical spectrum of frontal lobe seizures,^4^ delineated four distinct electroclinical subtypes based on detailed semiological analysis, with SMA and pre‐SMA involvement in both Group 1 and Group 2. The former is characterized by elementary motor signs, and the latter by nonintegrated gestural behaviors and proximal tonic posturing, reflecting the propagation or overlap with posterior prefrontal areas. Seizure semiology reflected the involvement of distributed epileptogenic networks, rather than isolated cortical regions. Overall, the study observed a rostro‐caudal gradient: seizures originating from rostral prefrontal areas produced the most integrated and complex behaviors, whereas those arising from posterior motor regions were limited to elementary motor signs. These findings emphasize the dynamic spatiotemporal evolution of seizure activity and its propagation through functional networks within the frontal lobe.

Despite these limitations, our systematic review allowed us to delineate the most frequent semiological features of SMA epilepsy.

## 5 CONCLUSION

The purpose of this study was to characterize the ictal semiology associated with SMA and pre‐SMA epilepsy, summarizing their anatomo‐clinical correlations in the context of presurgical evaluation. Asymmetric posturing has been consistently identified as the clinical sign most frequently associated with SMA epilepsy; however, the overall certainty of this association remains low, and no firm conclusions can be drawn regarding its diagnostic specificity. Additionally, our findings underscore the need to integrate semiology with neuroimaging and scalp EEG and to inform hypothesis‐driven implantation of Stereo‐EEG electrodes in order to optimize the surgical strategy in a region close to the motor cortex.

## CONFLICT OF INTEREST STATEMENT

None of the authors has any conflict of interest to disclose.

**Test yourself**

1. What is one of the main clinical challenges in localizing the epileptogenic zone in the supplementary motor area (SMA)?
  - The difficulty in localizing ictal and interictal abnormalities on scalp EEG
  - The semiological overlap with other types of frontal lobe seizures
  - The frequent need for invasive EEG (e.g., stereo‐EEG) to confirm epileptogenic zone
  - All of the above
2. According to the meta‐analysis, which ictal manifestation was most frequently associated with SMA and pre‐SMA epilepsy?
  - Fear and affective features
  - Complex gestural automatisms
  - Unilateral or bilateral asymmetric tonic posturing
  - Hyperkinetic movements
3. Which of the following symptoms is less frequent but well documented in SMA epilepsy?
  - Automatisms
  - Ictal speech inhibition
  - Hyperkinetic movements
  - Clonic jerks

*Answers may be found in the* *supporting information*.

## Supporting information


Data S1

Figures S1‐S12

Data S2

## Data Availability

The data that support the findings of this study are available from the corresponding author upon reasonable request.

## References

[1] Penfield W , Jasper H . Epilepsy and the functional anatomy of the human brain. Boston: Little, Brown and Company; 1954.

[2] Bancaud J , Talairach J . Functional organization of the supplementary motor area, data obtained by stereo‐E.E.G. Neurochirurgie. 1967;13:343–356. 4862665

[3] Fried I . Electrical stimulation of the supplementary sensorimotor area. Adv Neurol. 1996;70:177–185. 8615200

[4] Chauvel P , Trottier S , Vignal J‐P , Bancaud J . Somatomotor seizures of frontal lobe origin. Adv Neurol. 1992;57:185–232. 1543053

[5] Commission on Classification and Terminology of the International League Against Epilepsy . Proposal for revised clinical and electroencephalographic classification of epileptic seizures. Epilepsia. 1981;22:489–501. 10.1111/j.1528-1157.1981.tb06159.x 6790275

[6] Commission on Classification and Terminology of the International League Against Epilepsy . Proposal for revised classification of epilepsies and epileptic syndromes. Epilepsia. 1989;30(4):389–399. 10.1111/j.1528-1157.1989.tb05316.x 2502382

[7] Morris HH 3rd , Dinner DS , Lüders H , Wyllie E , Kramer R . Supplementary motor seizures: clinical and electroencephalographic findings. Neurology. 1988;38(7):1075–1082. 10.1212/wnl.38.7.1075 3386826

[8] Tachibana N , Shinde A , Ikeda A , Akiguchi I , Kimura J , Shibasaki H . Supplementary motor area seizure resembling sleep disorder. Sleep. 1996;19:811–816. 10.1093/sleep/19.10.811 9085490

[9] Meletti S , Rubboli G , Testoni S , Michelucci R , Cantalupo G , Stanzani‐Maserati M , et al. Early ictal speech and motor inhibition in fronto‐mesial epileptic seizures: a polygraphic study in one patient. Clin Neurophysiol. 2003;114(1):56–62. 10.1016/s1388-2457(02)00330-9 12495764

[10] Ikeda A , Sato T , Ohara S , Matsuhashi M , Yamamoto J , Takayama M , et al. “Supplementary motor area (SMA) seizure” rather than “SMA epilepsy” in optimal surgical candidates: a document of subdural mapping. J Neurol Sci. 2002;202(1–2):43–52. 10.1016/s0022-510x(02)00199-5 12220691

[11] Moher D , Shamseer L , Clarke M , Ghersi D , Liberati A , Petticrew M , et al. Preferred reporting items for systematic review and meta‐analysis protocols (PRISMA‐P) 2015 statement. Syst Rev. 2015;4(1):1. 10.1186/2046-4053-4-1 25554246 PMC4320440

[12] Engel J . Update on surgical treatment of the epilepsies. Summary of the second international Palm Desert conference on the surgical treatment of the epilepsies (1992). Neurology. 1993;43:1612–1617. 10.1212/wnl.43.8.1612 8102482

[13] Whiting PF . QUADAS‐2: a revised tool for the quality assessment of diagnostic accuracy studies. Ann Intern Med. 2011;155:529–536. 10.7326/0003-4819-155-8-201110180-00009 22007046

[14] Ryvlin P , Barba C , Bartolomei F , Baumgartner C , Brazdil M , Fabo D , et al. Grading system for assessing the confidence in the epileptogenic zone reported in published studies: a Delphi consensus study. Epilepsia. 2024;65:1346–1359. 10.1111/epi.17928 38420750

[15] Beniczky S , Trinka E , Wirrell E , Abdulla F , Al Baradie R , Alonso Vanegas M , et al. Updated classification of epileptic seizures: position paper of the international league against epilepsy. Epilepsia. 2025;66:1804–1823. 10.1111/epi.18338 40264351 PMC12169392

[16] Beniczky S , Tatum WO , Blumenfeld H , Stefan H , Mani J , Maillard L , et al. Seizure semiology: ILAE glossary of terms and their significance. Epileptic Disord. 2022;24:447–495. 10.1684/epd.2022.1430 35770761

[17] Freeman MF , Tukey JW . Transformations related to the angular and the square root. Ann Math Stat. 1950;21(4):607–611. 10.1214/aoms/1177729756

[18] Ohara S , Ikeda A , Kunieda T , Yazawa S , Taki J , Nagamine T , et al. Propagation of tonic posturing in supplementary motor area (SMA) seizures. Epilepsy Res. 2004;62(2‐3):179–187. 10.1016/j.eplepsyres.2004.09.002 15579306

[19] King DW , Smith JR . Supplementary sensorimotor area epilepsy in adults. Adv Neurol. 1996;70:285–291. 8615209

[20] McGonigal A , Bartolomei F , Regis J , Guye M , Gavaret M , Fonseca ATD , et al. Stereoelectroencephalography in presurgical assessment of MRI‐negative epilepsy. Brain. 2007;130(12):3169–3183. 10.1093/brain/awm218 17855377

[21] Sitthinamsuwan B , Usui N , Tottori T , Terada K , Kondo A , Matsuda K , et al. Seizures with tonic posturing: Semiologic difference between supplementary sensorimotor area (SSMA) origin and extra‐SSMA origin. Epilepsia. 2016;57(2):e39–e44. 10.1111/epi.13283 26660199

[22] Kellinghaus C , Lüders HO . Frontal lobe epilepsy. Epileptic Disord. 2004;6:223–262. 15634619

[23] Geier S , Bancaud J , Talairach J , Bonis A , Szikla G , Enjelvin M . The seizures of frontal lobe epilepsy. A study of clinical manifestations. Neurology. 1977;27:951–958. 10.1212/wnl.27.10.951 561909

[24] Barba C , Doglietto F , Policicchio D , Caulo M , Colicchio G . Unusual ipsilateral hyperkinetic automatisms in SMA seizures. Seizure. 2005;14:354–361. 15967684 10.1016/j.seizure.2005.04.011

[25] Geier S , Bancaud J , Talairach J , Bonis A , Enjelvin M , Hossard‐Bouchaud H . Automatisms during frontal lobe epileptic seizures. Brain. 1976;99:447–458. 1000282 10.1093/brain/99.3.447

[26] Bonini F , McGonigal A , Trébuchon A , Gavaret M , Bartolomei F , Giusiano B , et al. Frontal lobe seizures: from clinical semiology to localization. Epilepsia. 2014;55(2):264–277. 10.1111/epi.12490 24372328

[27] McLachlan R . The significance of head and eye turning in seizures. Neurology. 1987;37(10):1617–1619. 10.1212/wnl.37.10.1617 3658166

[28] Manford M , Fish DR , Shorvon SD . An analysis of clinical seizure patterns and their localizing value in frontal and temporal lobe epilepsies. Brain. 1996;119(Pt 1):17–40. 10.1093/brain/119.1.17 8624679

[29] McGonigal A . Frontal lobe seizures: overview and update. J Neurol. 2022;269:3363–3371. 35006387 10.1007/s00415-021-10949-0

[30] Tufenkjian K , Lüders HO . Seizure semiology: its value and limitations in localizing the epileptogenic zone. J Clin Neurol. 2012;8:243–250. 23323131 10.3988/jcn.2012.8.4.243 PMC3540282

[31] Wyllie E , Luders H , Morris HH , Lesser RP , Dinner DS . The lateralizing significance of versive head and eye movements during epileptic seizures. Neurology. 1986;36:606–611. 10.1212/wnl.36.5.606 3703259

[32] Lu J , Zhao Z , Zhang J , Wu B , Zhu Y , Chang EF , et al. Functional maps of direct electrical stimulation‐induced speech arrest and anomia: a multicentre retrospective study. Brain. 2021;144:2541–2553. 33792674 10.1093/brain/awab125 PMC8453410

[33] Penfield W , Welch K . The supplementary motor area in the cerebral cortex of man. Trans Am Neurol Assoc. 1949;74:179–184.

[34] Penfield W , Welch K . The supplementary motor area of the cerebral cortex. A clinical and experimental study. Arch Neurol Psychiatr. 1951;66:289–317. 14867993 10.1001/archneurpsyc.1951.02320090038004

[35] Fried I , Katz A , McCarthy G , Sass KJ , Williamson P , Spencer SS , et al. Functional organization of human supplementary motor cortex studied by electrical stimulation. J Neurosci. 1991;11:3656–3666. 1941101 10.1523/JNEUROSCI.11-11-03656.1991 PMC6575551

[36] Lim S , Dinner D , Pillay P , Lüders H , Morris H , Klem G , et al. Functional anatomy of the human supplementary sensorimotor area: results of extraoperative electrical stimulation. Electroencephalogr Clin Neurophysiol. 1994;91:193. 10.1016/0013-4694(94)90068-x 7522147

[37] Lüders HO , Dinner DS , Morris HH , Wyllie E , Comair YG . Cortical electrical stimulation in humans. The negative motor areas. Adv Neurol. 1995;67:115–129. 8848964

[38] Kovac S , Nachev P , Rodionov R , Scott C , McEvoy AW , Toms N , et al. Neck atonia with a focal stimulation‐induced seizure arising from the SMA: pathophysiological considerations. Epilepsy Behav. 2012;24(4):503–506. 10.1016/j.yebeh.2012.05.012 22749609

[39] Chassagnon S , Minotti L , Kremer S , Hoffmann D , Kahane P . Somatosensory, motor, and reaching/grasping responses to direct electrical stimulation of the human cingulate motor areas: clinical article. J Neurosurg. 2008;109(4):593–604. 10.3171/JNS/2008/109/10/0593 18826345

[40] Reutens D , Andermann F , Olivier A , Andermann E , Dubeau F . Unusual features of supplementary sensorimotor area epilepsy: cyclic pattern, unusual sensory aura, startle sensitivity, anoxic encephalopathy, and spontaneous remission. In: Lüders H , editor. Supplementary sensorimotor area. Philadelphia, PA: Lippincott‐Raven; 1996. 8615211

[41] Canuet L , Ishii R , Iwase M , Kurimoto R , Ikezawa K , Azechi M , et al. Cephalic auras of supplementary motor area origin: an ictal MEG and SAM(g2) study. Epilepsy Behav. 2008;13(3):570–574. 10.1016/j.yebeh.2008.05.013 18585961

[42] Tao Y , Guojun Z , Yuping W , Lixin C , Wei D , Yongjie L . Surgical treatment of patients with drug‐resistant hypermotor seizures. Epilepsia. 2010;51(10):2124–2130. 10.1111/j.1528-1167.2010.02590.x 20491874

[43] Nitta N , Usui N , Kondo A , Tottori T , Terada K , Araki Y , et al. Semiology of hyperkinetic seizures of frontal *versus* temporal lobe origin. Epileptic Disord. 2019;21(2):154–165. 10.1684/epd.2019.1047 31010798

[44] Ikeda A , Nagamine T , Kunieda T , Yazawa S , Ohara S , Taki W , et al. Clonic convulsion caused by epileptic discharges arising from the human supplementary motor area as studied by subdural recording. Epileptic Disord. 1999;1(1):21–26. 10937128

[45] Souirti Z , Landré E , Mellerio C , Devaux B , Chassoux F . Neural network underlying ictal pouting (“chapeau de gendarme”) in frontal lobe epilepsy. Epilepsy Behav. 2014;37:249–257. 10.1016/j.yebeh.2014.07.009 25108117

[46] Leitinger M , Höfler J , Deak I , Kalss G , Rohracher A , Kuchukhidze G , et al. “Chapeau de gendarme”—a frontomesial ictal sign? Epilepsy Behav. 2015;44:258–259. 10.1016/j.yebeh.2014.12.033 25669591

