# Supplementary material for: Ictal semiology in supplementary motor area and pre‐supplementary motor area epilepsy: A systematic review and meta‐analysis
Source: Epileptic Disord. 2025 Nov 24;28(1):33–42. doi: 10.1002/epd2.70137 (PMC12964175; doi:10.1002/epd2.70137)
Supplement: Supplementary file 1 — Data S1 [file EPD2-28-33-s001.docx]

**Supplementary Material - Methods**

1. **Data extraction**

We evaluated the risk of bias of each publication using a QUADAS2-adapted ^1^ assessment at the level of each selected publication as follows:

**Risk of selection bias**

Was a consecutive or random sample of patients enrolled?

Was a case-control design used?

Did the study avoid inappropriate exclusions?

Could the selection of patients have introduced bias?

**Risk of assessment bias**

Was semiology interpreted blinded to other data?

1. **Reliability of the reference standard**

We further assessed our level of confidence in the reported epileptogenic zone according to a recently developed method.^14^ The latter is based on the availability and findings from MRI, intracerebral EEG and post-operative outcome, and distinguishes four levels of evidence (very high, high, moderate and low) defined as follows:

1) “very high” confidence in the reported EZ for patients with Engel class IA after at least one year of post-operative follow-up;

2) “high” confidence in the reported EZ for patients with either: i) a well delineated focal lesion suspected to represent at least part of the EZ (according to the authors of the publication), or ii) a well delineated EZ according to all available iEEG data (according to the authors of the publication), or iii) an Engel class I (but not specified IA) after at least one year of post-operative follow-up;

3) “moderate” confidence in the reported EZ for patients with MRI signs of hippocampal sclerosis or atrophy suspected to be at least part of the EZ;

4) “low” confidence in the reported EZ for patients whose MRI would be normal or show multilobar, multifocal or poorly delineated lesion, or with a poorly delineated EZ according to all available iEEG data (according to the authors of the publication), or an Engel class II-IV post-operative outcome provided the entire EZ has been entirely removed. Surgical failure in patients whose suspected EZ would not have been fully removed would not be considered for grading.

If several of the above items were available and provided different levels of confidence, that associated with the post-operative outcome prevailed over the iEEG and MRI findings, while iEEG conclusions would prevail over MRI findings.

For each selected paper, we indicated the proportions of patients falling into each of the above-mentioned categories.

1. **Overall summary of evidence**

The summary of evidence was eventually assessed using the GRADE system, according to the following categories of the level of evidence:

- Very low reliability: The true effect is probably markedly different from the estimated effect;
- Low reliability: The true effect might be markedly different from the estimated effect;
- Moderate reliability: The authors believe that the true effect is probably close to the estimated effect;
- High reliability: The authors have a lot of confidence that the true effect is similar to the estimated effect.
