# Supplementary material for: Ictal semiology in supplementary motor area and pre‐supplementary motor area epilepsy: A systematic review and meta‐analysis
Source: Epileptic Disord. 2025 Nov 24;28(1):33–42. doi: 10.1002/epd2.70137 (PMC12964175; doi:10.1002/epd2.70137)
Supplement: Supplementary file 2 — Figures S1‐S12 [file EPD2-28-33-s002.docx]

**Supplemental figures 1-12:** Forrest plots summarizing the pooled prevalence of semiological features observed in patients with seizures originating from the Supplementary Motor Area (SMA). Each plot represents a distinct semiological sign.


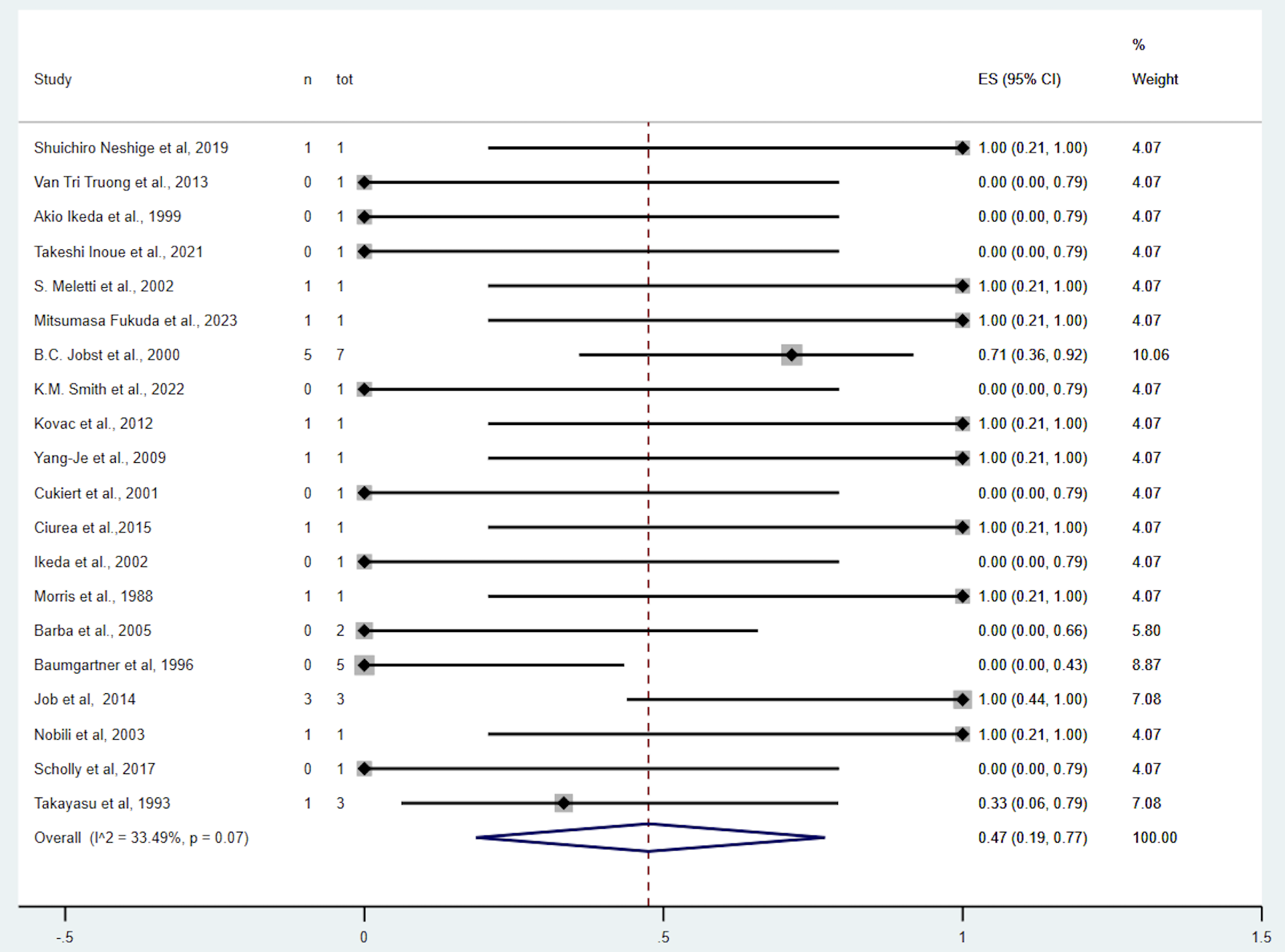


Figure 1: Meta-­analysis showing the prevalence of patients with Asymmetric Tonic Posturing. Asymmetric tonic posture occurs in approximately half of the population. There is wide heterogeneity among included studies (from 0% to 100%).

*
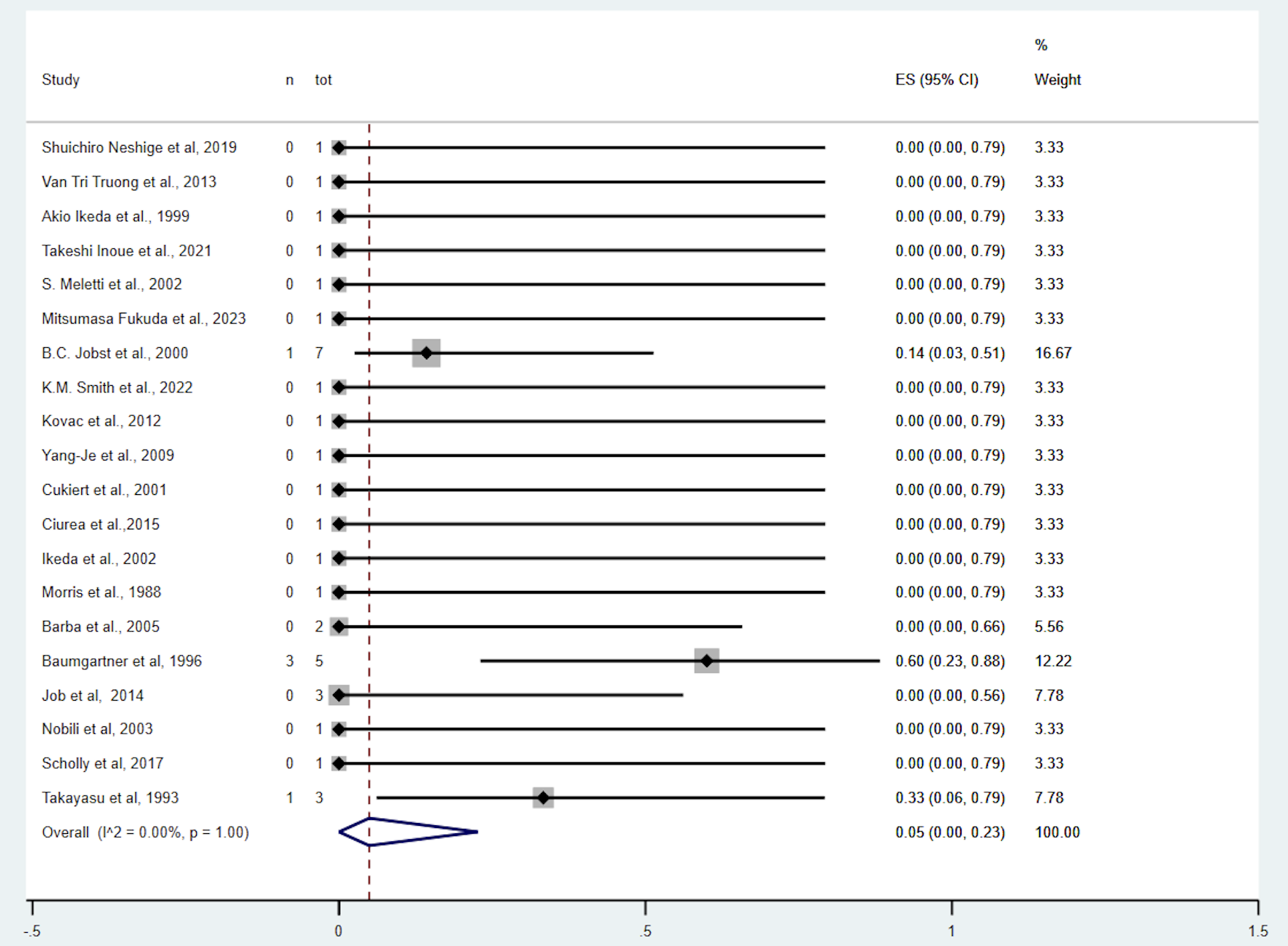
*

Figure 2: Meta-­analysis showing the prevalence of patients with Symmetric Tonic Posturing. Symmetric Tonic Posturing is present in only 5% of subjects with SMA. This semiological feature is not a good diagnostic marker of SMA

*
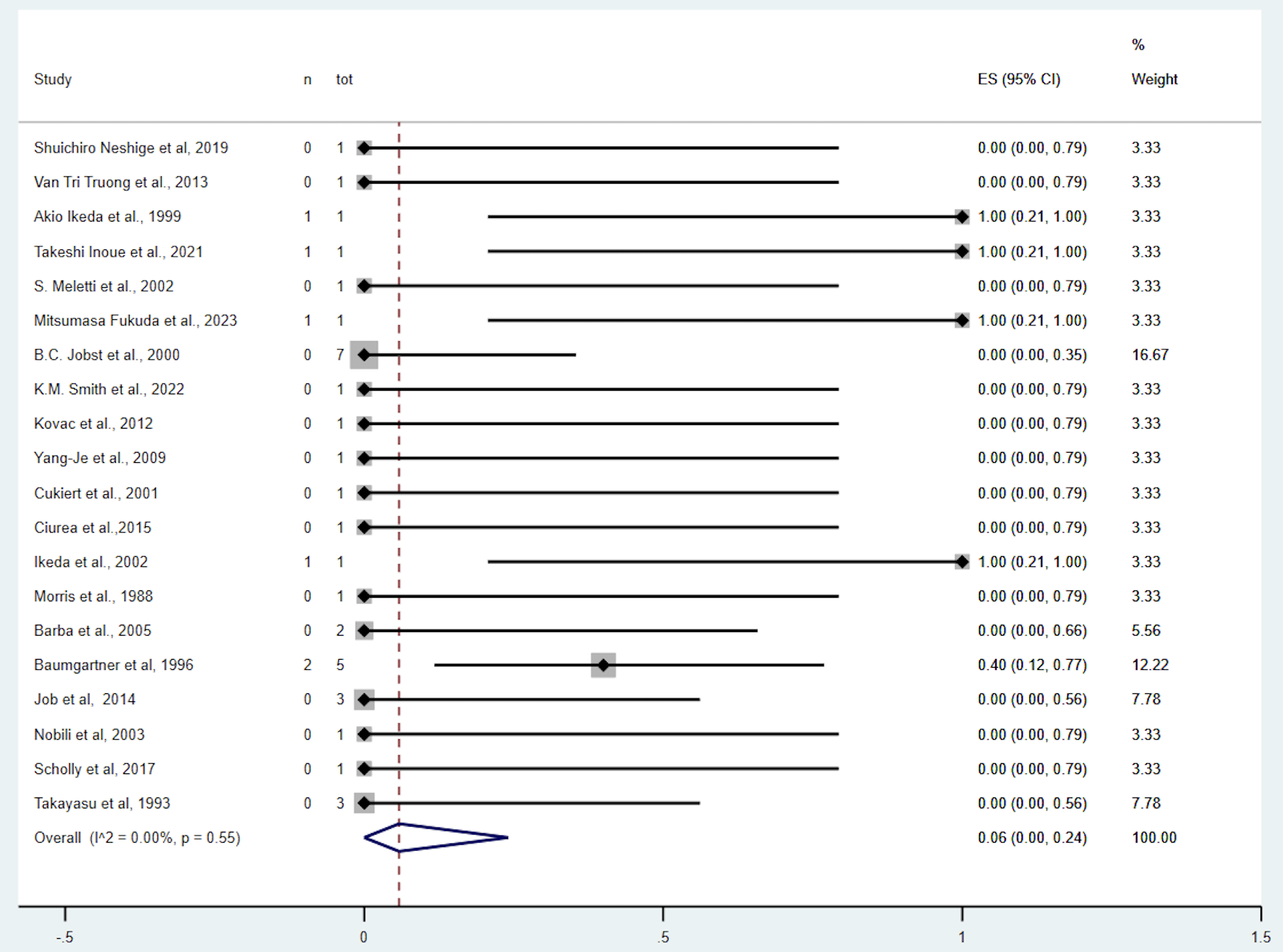
*

Figure 3: Meta-­analysis showing the prevalence of patients with Elementary motor signs

Elementary motor signs is present in only 6% of subjects with SMA. This semiological feature is not a good diagnostic marker of SMA

*
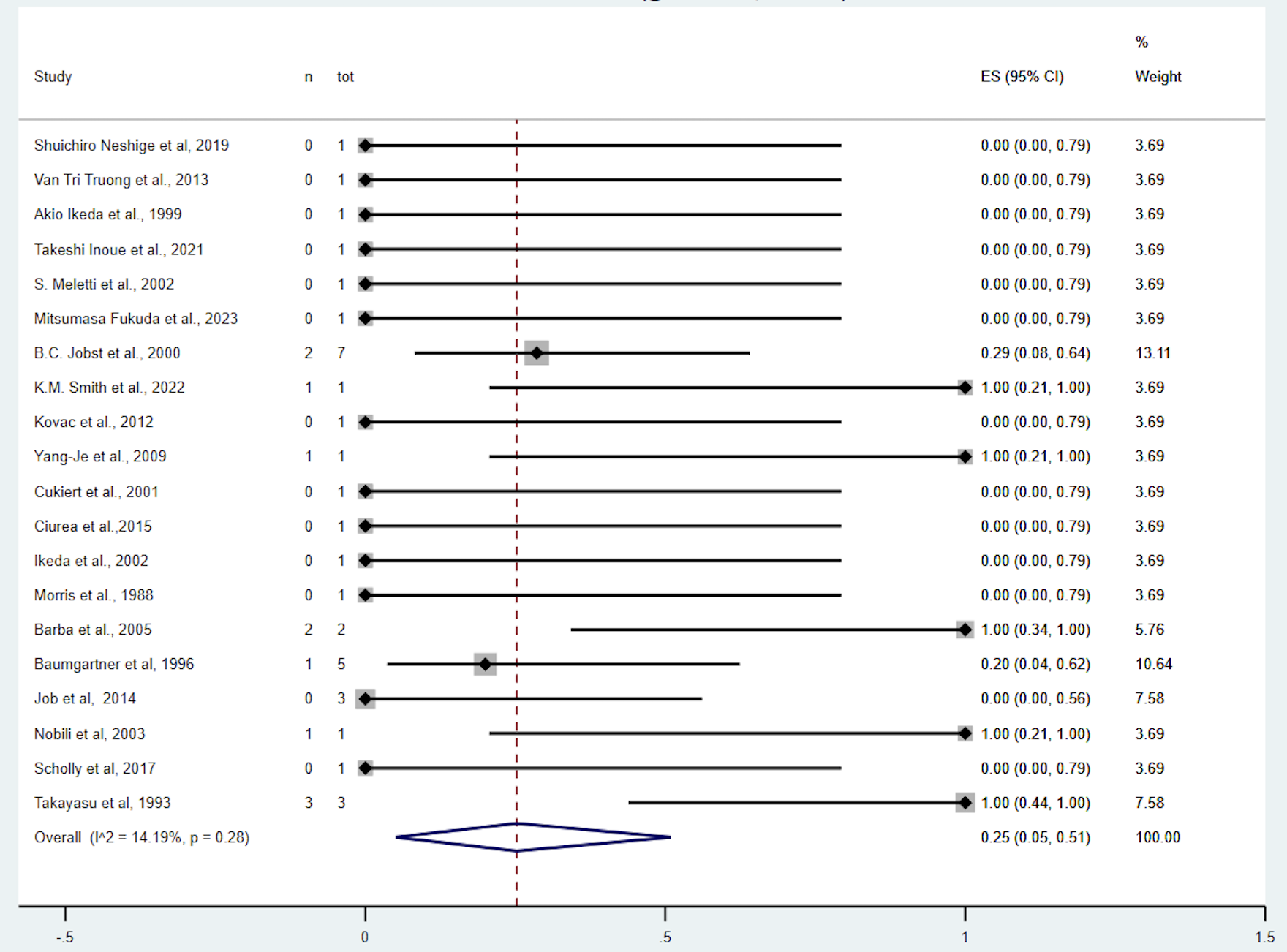
*

Figure 4: Meta-­analysis showing the prevalence of patients with Automatisms

Automatisms are present in about 1 of 4 patients with SMA.

*
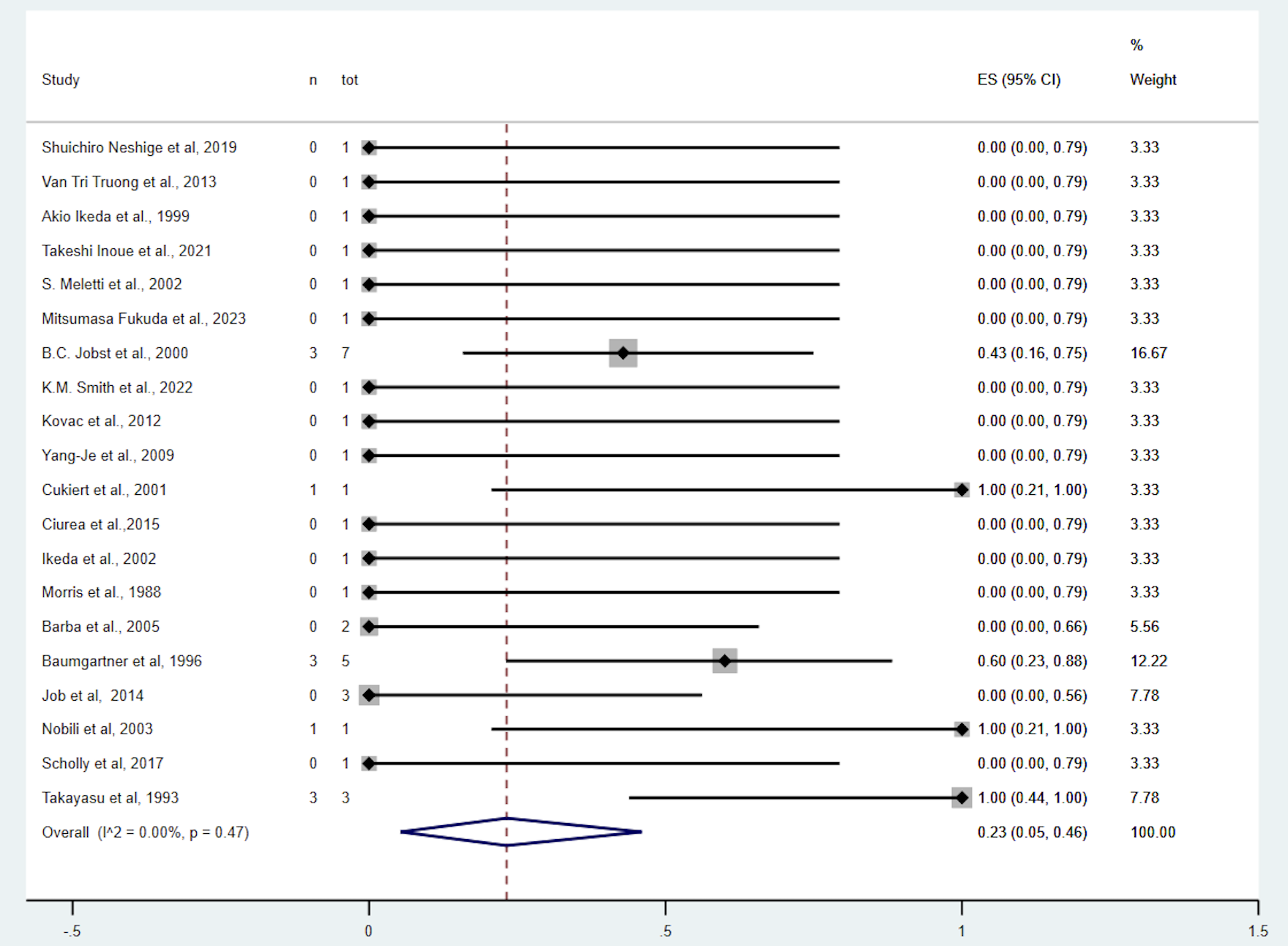
*

Figure 5: Meta-­analysis showing the prevalence of patients with Head and/or eye Version

Head and/or eye Version is present in 23% of patients with SMA.

*
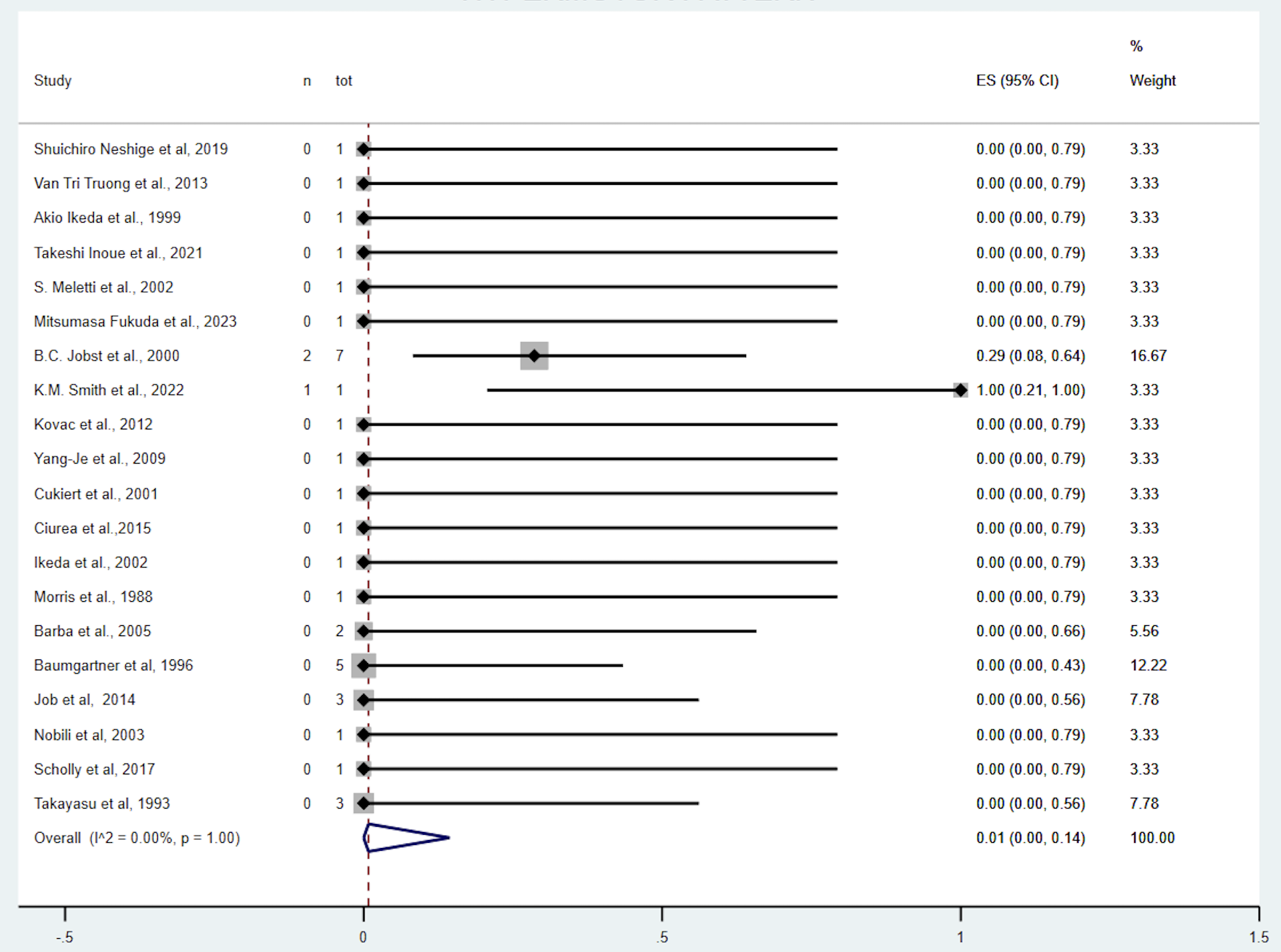
*

Figure 6: Meta-­analysis showing the prevalence of patients with Hyperkinetic Pattern

Hyperkinetic pattern is extremely rare in patients with SMA.

*
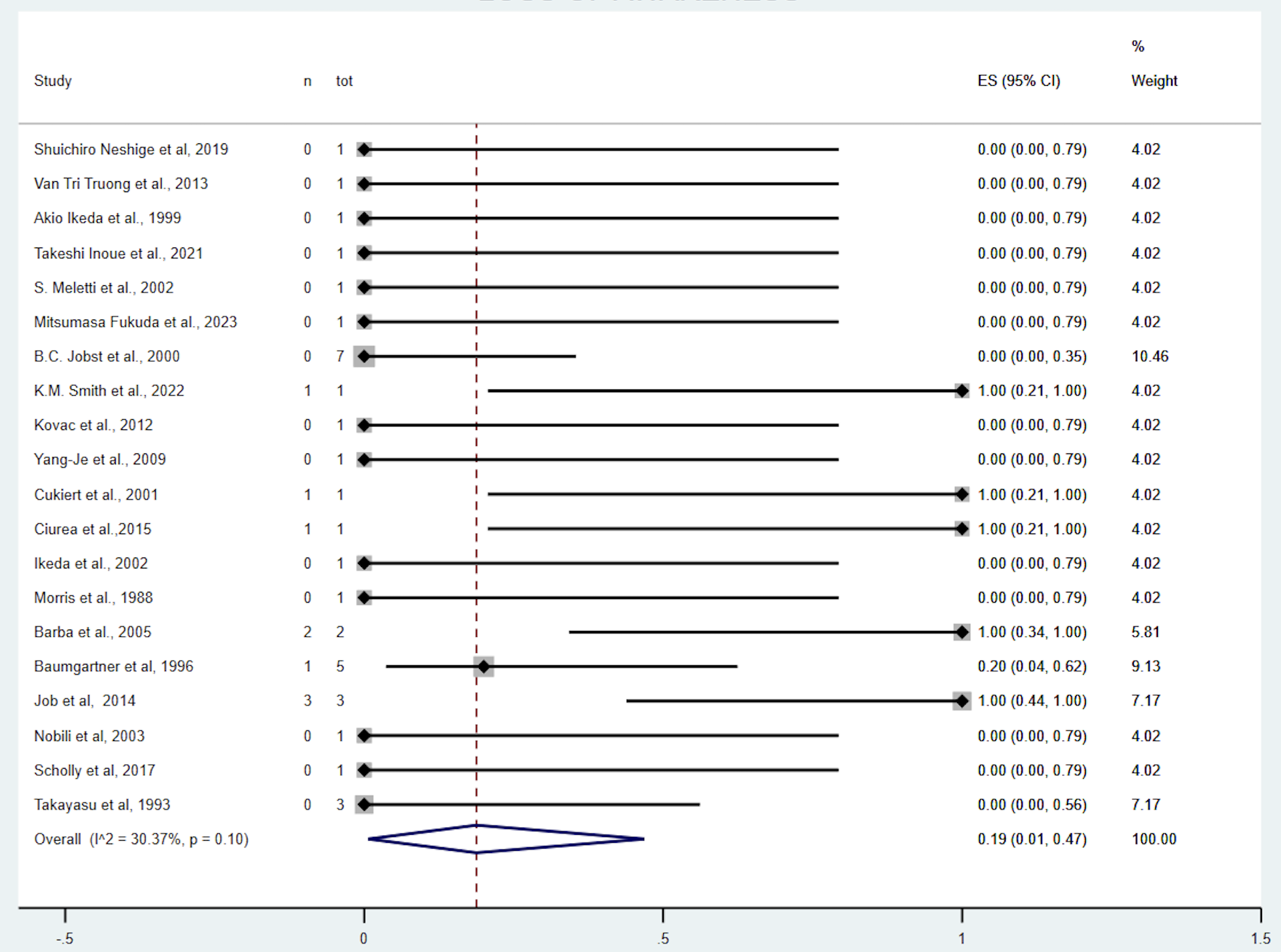
*

Figure 7: Meta-­analysis showing the prevalence of patients with impaired awareness

Impaired Awareness is present in about 1 of 5 patients with SMA. The results of the included studies are slightly heterogeneous (I2=30.37%; p=0.10)

*
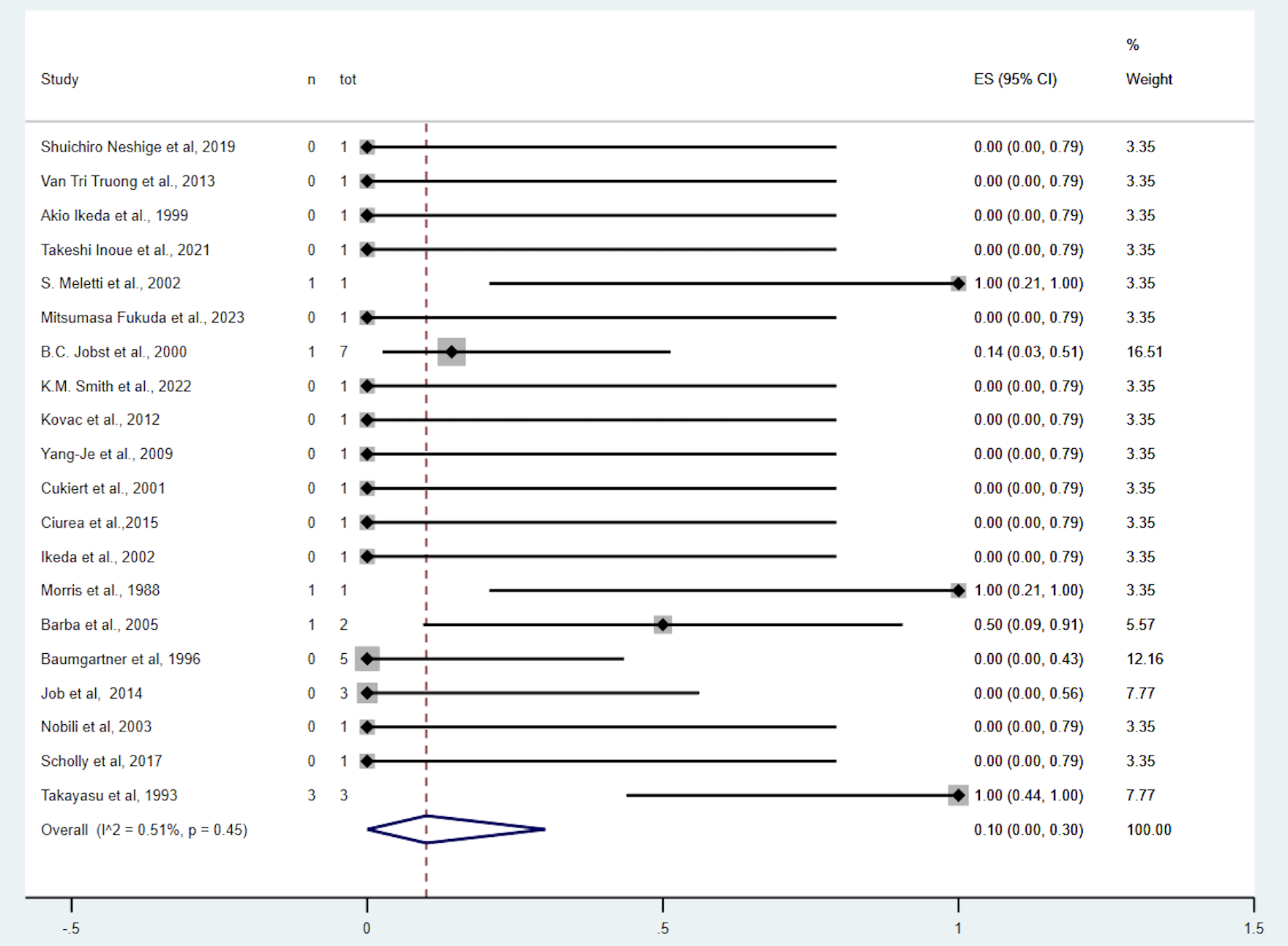
*

Figure 8: Meta-­analysis showing the prevalence of patients with Speech Inhibition

Speech Inhibition occurs in about 1 of 10 patients with SMA. The results of the included studies are homogeneous.(I2=0.51%; p=0.45)

*
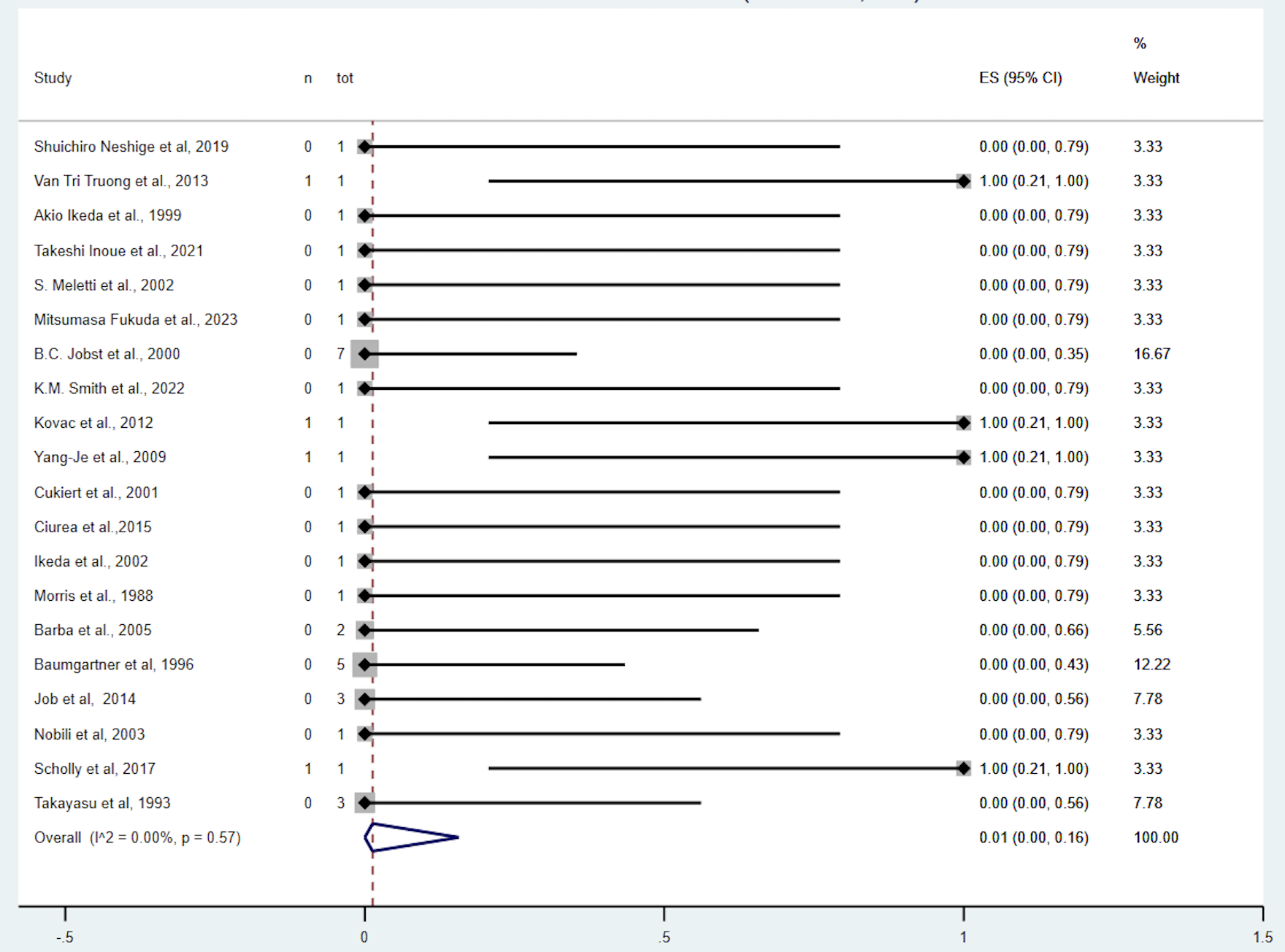
*

Figure 9: Meta-­analysis showing the prevalence of patients with Other Negative Phenomena

Other Negative Phenomena are extremely rare in patients with SMA. The results of the included studies are homogeneous (I2=0%, p=0.57)

*
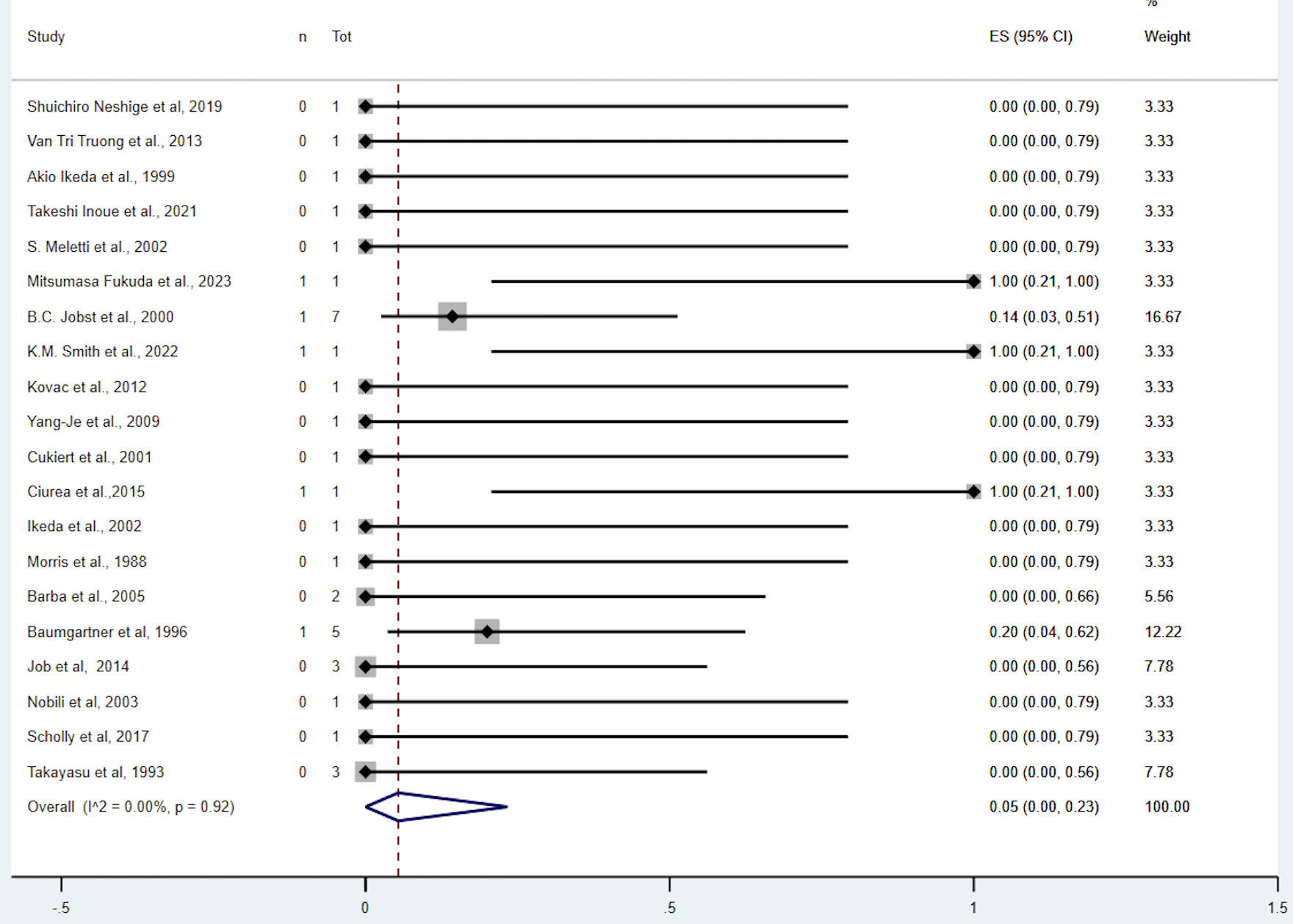
*

Figure 10: Meta-­analysis showing the prevalence of patients with Grimacing

The prevalence of grimacing is approximately 5% in patients with SMA. The results of the included studies are highly homogeneous (I2=0%, p=0.92)

*
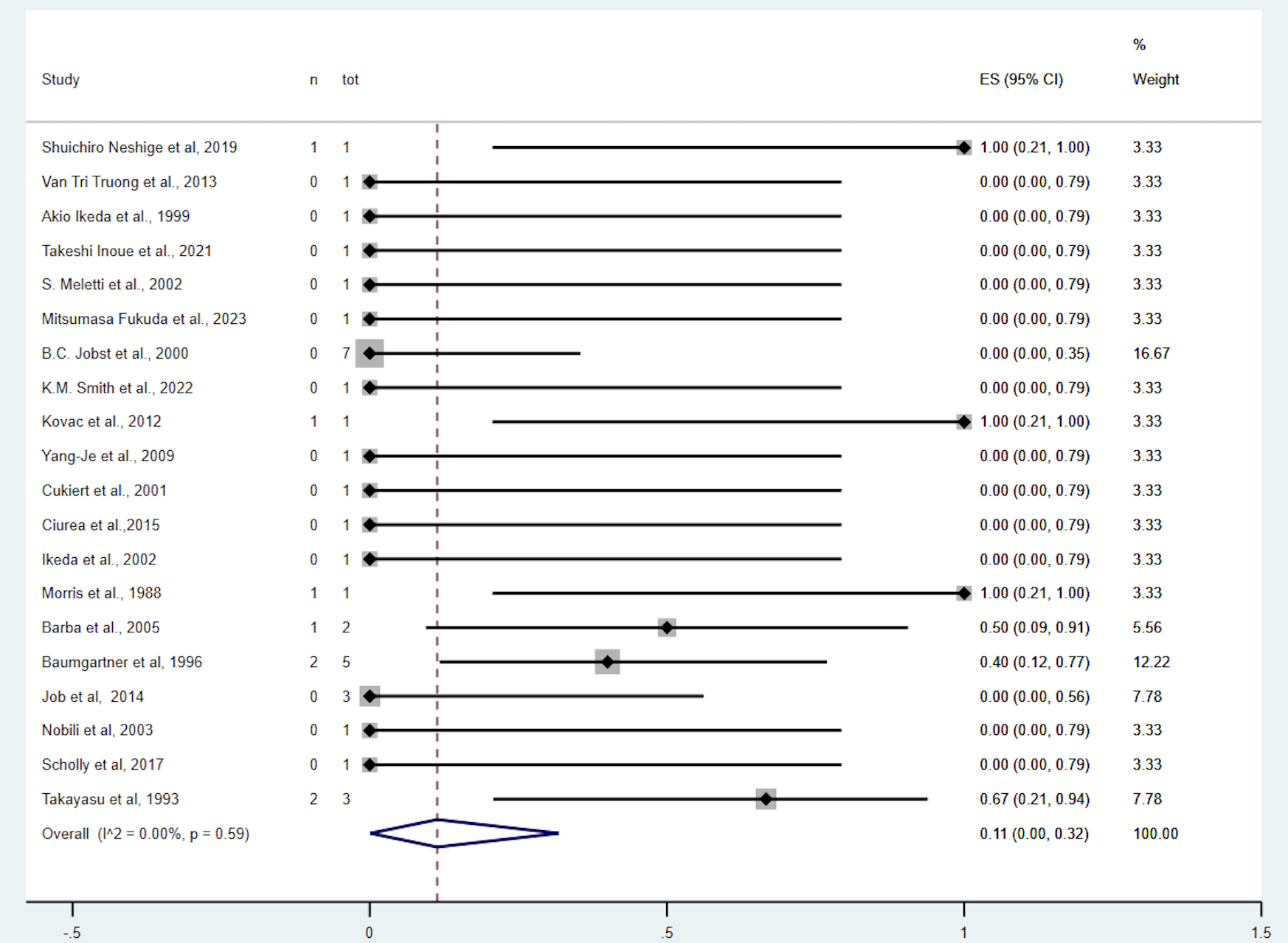
*

Figure 11: Meta-­analysis showing the prevalence of patients with Sensory Phenomena

Sensory Phenomena occur in 11% of patients with SMA. No heterogeneity is detected (I2=0%, p=0.59)

*
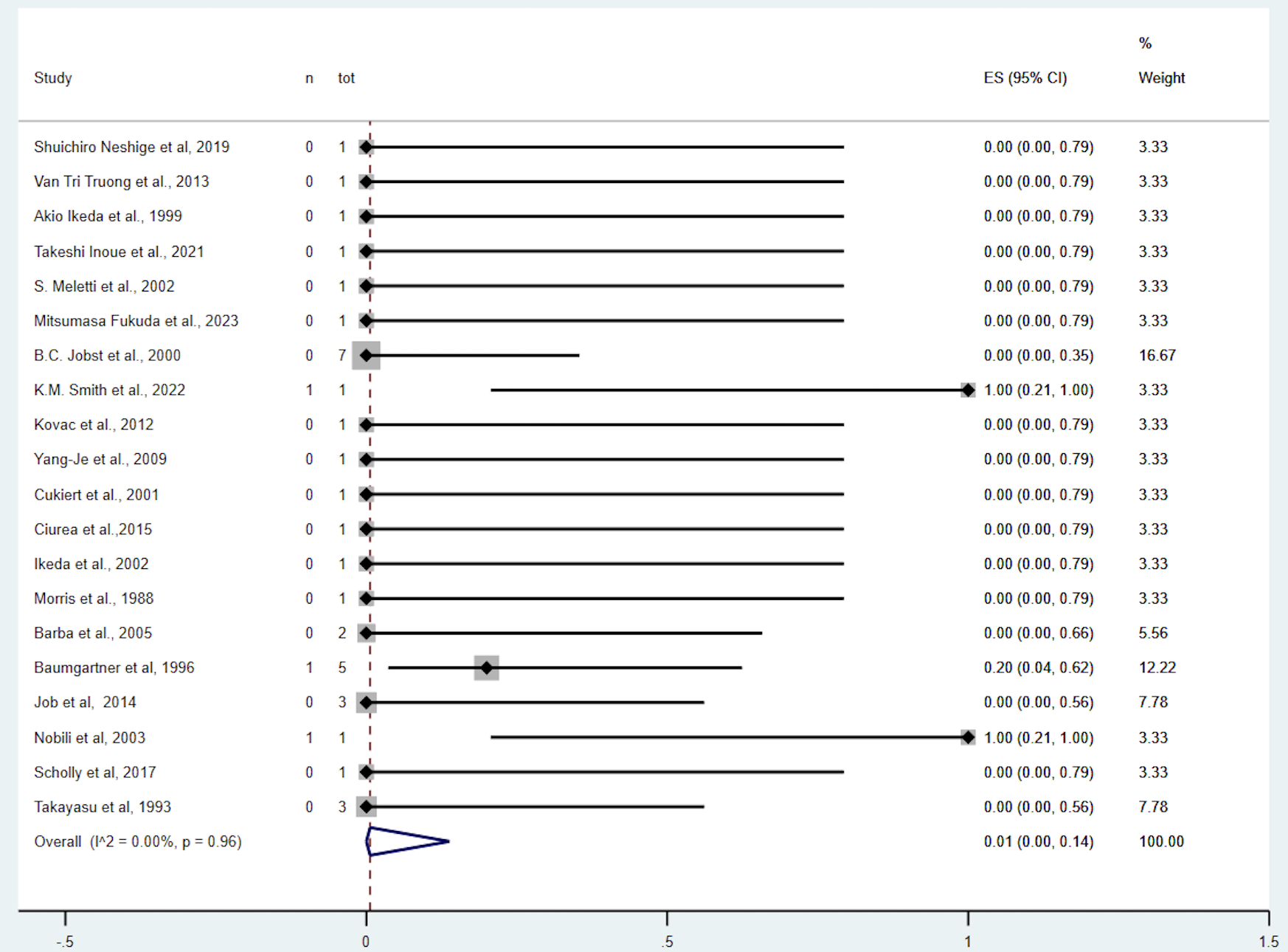
*

Figure 12: Meta-­analysis showing the prevalence of patients with Affective Phenomena

Affective Phenomena are extremely rare in patients with SMA. The results of the included studies are higly homogeneous (I2=0%, p=0.96)
