# Supplementary material for: Ictal semiology in supplementary motor area and pre‐supplementary motor area epilepsy: A systematic review and meta‐analysis
Source: Epileptic Disord. 2025 Nov 24;28(1):33–42. doi: 10.1002/epd2.70137 (PMC12964175; doi:10.1002/epd2.70137)
Supplement: Supplementary file 3 — Data S2 [file EPD2-28-33-s003.docx]

1. Answer: D.

2. Answer: C.

3. Answer: B.
